# The reproducibility of measuring maximum abdominal aortic aneurysm diameter from ultrasound images

**DOI:** 10.1186/s13089-021-00211-z

**Published:** 2021-02-26

**Authors:** Evan O. Matthews, Jenna Pinchbeck, Kylie Elmore, Rhondda E. Jones, Joseph V. Moxon, Jonathan Golledge

**Affiliations:** 1 Queensland Research Centre for Peripheral Vascular Disease, College of Medicine and Dentistry, James Cook University, Townsville, QLD 4811 Australia; 2 Department of Vascular and Endovascular Surgery, Townsville University Hospital, Townsville, QLD 4812 Australia; 3 Australian Institute for Tropical Health and Medicine, James Cook University, Townsville, QLD 4811 Australia

**Keywords:** Abdominal aortic aneurysm, Ultrasound, Reproducibility

## Abstract

**Background:**

Accurate repeat assessment of the diameter of an abdominal aortic aneurysm (AAA) is important. This study investigated the reproducibility of different methods of measuring AAA diameter from ultrasound images.

**Methods:**

Fifty AAA patients were assessed by ultrasound. Maximum AAA diameter was measured independently by three trained observers on two separate occasions using a standardised protocol. Five diameters were measured from each scan, three in the anterior–posterior (AP) and two in the transverse (TV) plane, including inner-to-inner (ITI), outer-to-outer (OTO) and leading edge-to-leading edge (LETLE). Intra- and inter-observer reproducibility were reported as reproducibility coefficients. Statistical comparison of methods was performed using linear mixed effects models.

**Results:**

Intra-observer reproducibility coefficients (AP LETLE 2.2 mm; AP ITI 2.4 mm; AP OTO 2.6 mm) were smaller than inter-observer reproducibility coefficients (AP LETLE 4.6 mm: AP ITI 4.5; and AP OTO 4.8 mm). There was no statistically significant difference in intra-observer reproducibility of three types of measurements performed in the AP plane. Measurements obtained in the TV plane had statistically significant worse intra-observer reproducibility than those performed in the AP plane.

**Conclusions:**

This study suggests that the comparison of maximum AAA diameter between repeat images is most reproducibly performed by a single trained observer measuring diameters in the AP plane.

## Background

Approximately 2% of men and 0.5% of women aged > 65 years develop an abdominal aortic aneurysm (AAA) [1, 2]. Maximum AAA diameter is the most established predictor of AAA growth and rupture and is used in clinical practice to guide decision-making [3–5]. Current guidelines recommend considering AAA repair when the maximum diameter is ≥ 55 mm in men and ≥ 50 mm in women [6, 7]. Management of smaller asymptomatic AAAs is by surveillance with repeat imaging performed at intervals to monitor maximum AAA diameter. Furthermore, there is growing interest in the identification of drug therapies to slow AAA growth [8, 9]. Trials testing potential drugs usually assess outcome by monitoring maximum AAA diameter growth over time [10]. Reproducible methods to measure AAA diameter are therefore of both clinical and research importance.

In clinical practice and most previous clinical trials ultrasound imaging has been used to estimate maximum AAA diameter [6, 10]. Despite the importance of accurate determination of AAA diameter, measurement protocols are often incompletely reported and vary in both plane of acquisition and calliper placement [11]. The United Kingdom Small Aneurysm Trial reported maximal anterior–posterior outer-to-outer diameter (OTO) from ultrasound [12, 13]. AAA screening programmes have used a variety of different methods to measure AAA diameter on ultrasound, including the inner-to-inner (ITI) or leading edge-to-leading-edge (LETLE) methods of calliper placement [14, 15]. Disparate methods of calliper placement has been reported to cause differences of up to 5 mm in maximal AAA diameter with implications for decision-making regarding surgical repair and surveillance intervals which could impact on patient care [16, 17]. A number of theoretical principles have been cited as justification for different calliper selection, but no standardised method has been ubiquitously adopted. Three recent studies have compared these three methods of calliper placement, but had inconsistent findings [16–18].

A key aspect in choosing a method of measurement is its repeatability or reproducibility. This is particularly important for AAA diameter as this is commonly remeasured at intervals during which only small changes occur. This study aimed to compare the inter- and intra-observer repeatability of five different methods of measuring AAA diameter using distinct measurement planes and calliper placement.

## Methods

### Study design and participant recruitment

Participants were recruited from The Department of Vascular and Endovascular Surgery at Townsville University Hospital [19]. All participants provided written informed consent for inclusion and ethics approval was obtained from The Townsville Hospital and Health Service Human Research Ethics Committee (13/QPCH/16). A convenience sample size of 50 participants was used in concordance with previous similar studies [16, 17, 20]. Participants met the following inclusion criteria: (1) a small infra-renal AAA measuring 30–55 mm in maximal diameter and (2) an ultrasound performed by one of three experienced vascular sonographers within the Townsville University Hospital Vascular Laboratory using a standardised protocol. Patients with a history of previous abdominal aortic surgery were excluded. At recruitment an interview and physical examination was conducted to collect relevant medical history and clinical measurements. Ischaemic heart disease, hypertension and diabetes were defined as a previous diagnosis or treatment of these conditions by a qualified medical physician. Stroke was defined as a documented history of ischaemic or haemorrhagic stroke. The presence of aneurysms at other sites was determined through documented history or relevant medical imaging. Brachial blood pressure, waist circumference and body mass index were measured as previously described [21].

### Ultrasound imaging

All ultrasound scans were performed using a Phillips iu22 machine (Phillips Medical Systems, United States) with a C5-1 MHz general purpose curvilinear abdominal transducer by one of three experienced sonographers between December 2013 and December 2015. Each sonographer was a formally trained and registered sonographer with specialised experience in vascular ultrasound. Each participant was fasted for 12 h prior to their ultrasound scan to minimise interference from bowel gas. All participants were scanned in the supine position. The abdominal aorta was examined in both the transverse and sagittal planes to identify infra-renal landmarks, vessel tortuosity and obliqueness. Static ultrasound images in the transverse plane acquired in systole were obtained at the point of maximal dilation of the infra-renal abdominal aorta perpendicular to the central vessel line. The images were then centrally stored in accordance with health service policy.

### Measurement protocol

**Fig. 1:**
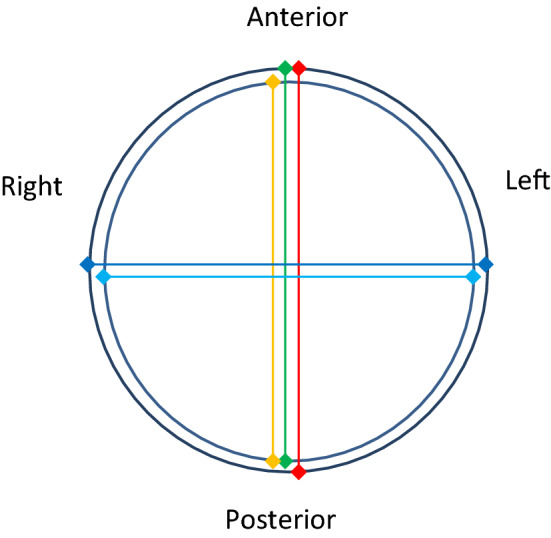
Illustration of a transverse view of an abdominal aortic aneurysm with the participant in the supine position. Figure shows ideal placement of callipers for ITI ( / ), LETLE ( ), OTO ( / ) in both anterior–posterior and transverse direction. OTO: outer-to-outer; LETE: leading edge-to-leading edge; ITI: inner-to-inner

Three observers were trained to measure maximal AAA diameter on static ultrasound images using a predefined protocol. Observers were selected based on pre-existing knowledge of aortic anatomy, cardiovascular physiology and imaging. Observer one was a qualified vascular sonographer with extensive experience in acquiring and interpreting ultrasound imaging of the aorta. Observer two was a clinical medical student with previous experience measuring AAA growth on computerised tomographic angiography scans. Observer three was a research worker and exercise physiologist with extensive experience in interpreting static ultrasound images of the abdominal aorta. A set measurement protocol was developed in consultation between a vascular sonographer, vascular surgeon and researcher. Five measurements were performed to assess AAA diameter. Three in the anterior–posterior (OTO, ITI and LETLE) and two (OTO and ITI) in the transverse plane (Fig. 1). Observer training involved both theoretical discussion and practical demonstration of the measurement protocol. Observers then measured a separate series of ten static ultrasound scan images independently before a consensus discussion was conducted on calliper placement between observers. Static ultrasound images were imported as DICOM images to the OsiriX Lite 32-bit version (Pixmeo, Geneva, Switzerland) software for analysis. To avoid bias, only static images where the sonographer placed measurement callipers had been omitted were included in this study. Prior to each measurement, each observer measured a 10-mm marked interval on a 100-mm scale to ensure accurate calibration of callipers. Each observer independently measured identical static images from the 50 participants and were blinded to the other observers’ measurements. Each observer repeated measurements 1 week later blinded to their earlier results.

### Data analysis

Data analyses were performed in SPSS Version 23.0 (IBM SPSS Inc., Chicago, IL, United States) and R (Foundation for Statistical Computing, Vienna, Austria) with assistance from an expert statistician (REJ). Demographic data were reported as count (%) for dichotomous data and median (inter-quartile range 25th to 75th centile) for continuous data.

### Intra- and inter-observer reproducibility

The mean and standard deviation (SD) of the differences between measurement one and two were calculated for each observer. The SD of the differences were then multiplied by 1.96 to obtain the reproducibility coefficient, or 95% limits of agreement (LOA), for each individual observer. The MethComp package in R was used to combine data from all three observers to calculate the pooled reproducibility coefficient for each individual method. Each scan–observer combination was treated as an individual item*.* Linear mixed effects models were used to formally test for a significant difference in repeatability between each individual method by comparing squared mean differences from a reference method (anterior–posterior ITI). Observers and subjects were both treated as random effects and mean difference and calliper placement method as fixed effects. The overall mean difference between ITI, LETLE and OTO for each scan was also calculated.

## Results

The risk factors of the included patients are reported in Table 1. Median maximum diameter of the included patients varied by up to 8.3 mm depending on the measurement method used (Table 1).

**Table 1:** Patient characteristics

Characteristic	Number (%)
Male	38 (74%)
Age	72 (68–77)
AAA AP OTO diameter (mm)	41.8 (37.4–44.9)
AAA AP LETLE diameter (mm)	38.2 (34.4–41.7)
AAA AP ITI diameter (mm)	35.8 (31.9–38.8)
AAA TV OTO diameter (mm)	44.1 (38.8–48.4)
AAA TV ITI diameter (mm)	37.3 (33.0–41.3)
Systolic blood pressure*	138 (127–148)
Diastolic blood pressure*	77 (70–82)
Hypertension	42 (84%)
Diabetes	11 (22%)
Ischaemic heart disease	34 (68%)
Stroke	8 (16%)
Aneurysm at another site	11 (22%)
Waist circumference (cm)	105.5 (97–114)
Body mass index (kg.m^−2^)	28.5 (26.1–32.9)

Continuous variables are presented as median (inter-quartile range)

Categorical variables are presented as count (percent)

AP: anterior–posterior; TV: transverse; OTO: outer-to-outer; LETE: leading edge-to-leading edge; ITI: inner-to-inner

^*^Four values missing

### Intra-observer reproducibility

The intra-observer reproducibility coefficients for each individual observer are shown in Table 2. There was notable variation in reproducibility coefficient between each individual method and each individual observer. Scans obtained in the anterior–posterior plane and with LETLE calliper placement had the lowest overall intra-observer reproducibility coefficient (± 2.2 mm), but this was not statistically significantly different from ITI and OTO calliper placement in the same plane. All three observers consistently measured images in the transverse plane with a poorer repeatability as highlighted by the poorer overall intra-observer reproducibility coefficient for both ITI (± 4.9 mm) and OTO (± 5.6 mm) calliper placement. Measurements in the transverse plane (ITI *P* = 0.001; OTO *P* < 0.001) were significantly less reproducible than those measured in the AP plane (Table 2).

**Table 2:** Intra-observer reproducibility coefficients

Anatomical plane	Calliper placement	Reproducibility coefficient				Comparison between methods		
		Observer 1 (± mm)	Observer 2 (± mm)	Observer 3 (± mm)	Overall (± mm)	Mean difference squared	Standard error	*P*-value
AP	ITI	2.22	1.68	2.65	2.35	1.38	1.19	Reference
AP	LETLE	2.17	1.89	2.37	2.16	-0.17	1.43	0.906
AP	OTO	2.31	2.53	2.50	2.60	0.31	1.43	0.830
Transverse	ITI	4.76	4.91	4.83	4.90	4.63	1.44	0.001
Transverse	OTO	5.62	4.74	5.51	5.62	6.49	1.44	< 0.001

ITI: Inner-to-inner; OTO: outer-to-outer; LETLE: leading edge-to-leading edge; AP: anterior–posterior

### Inter-observer reproducibility

The inter-observer reproducibility coefficient for each method is shown in Table 3. The inter-observer reproducibility coefficients were poorer than the corresponding intra-observer reproducibility coefficient for each method. The inter-observer reproducibility was poorest for images measured transversely (Table 3).

**Table 3:** Inter-observer reproducibility coefficients

Patient position	Anatomical direction	Method	Reproducibility coefficient (± mm)	Mean diameter difference from reference method (mm)
Supine	AP	ITI	4.47	Reference
Supine	AP	LETLE	4.59	2.69
Supine	AP	OTO	4.82	5.52
Supine	Transverse	ITI	6.02	Reference
Supine	Transverse	OTO	6.22	6.40

ITI: inner-to-inner; OTO: outer-to-outer; LETLE: leading edge-to-leading edge; AP: anterior–posterior

## Discussion

This study examined the influence of alternative methods of measurement of AAA diameter under conditions typically required in clinical trials. There was no statistically significant difference between alternative methods of measurement where calliper placement was in line with probe positioning (anterior–posterior for supine position). Measurements obtained perpendicular to the probe (transverse in the supine position) have been reported to be less repeatable due to poorer resolution of the lateral vessel walls [22]. This study supports this finding with statistically significant worse intra-observer reproducibility in both ITI and OTO measurements obtained in the transverse direction compared to those measured anterior–posterior.

Sixteen previous studies reporting the reproducibility of abdominal aortic measurements with ultrasound were identified [14, 16–18, 20, 23–33]. There was marked variation in reproducibility coefficients for both intra-observer (range ± 0.9 mm to ± 4.0 mm) and inter-observer (range ± 1.7 mm to ± 12.6 mm) repeatability. Gurtelschmid et al. reported better inter-observer reproducibility coefficients in anterior–posterior LETLE (± 4.0 mm) and anterior–posterior ITI (± 4.6 mm) calliper placement when compared with anterior–posterior OTO (± 5.3 mm) calliper placement. Borgbjerg et al. reported similar findings with better inter-observer reproducibility coefficients with anterior–posterior LETLE (± 3.8 mm) and anterior–posterior ITI (± 3.9 mm) calliper placement compared with anterior–posterior OTO (± 5.2 mm) [16, 18]. These findings are in contrast to those of Chui *et* al. who reported no statistical difference in reproducibility coefficients between these three methods (anterior–posterior LETLE ± 3.5 mm; anterior–posterior ITI ± 4.8 mm; anterior–posterior OTO ± 3.4 mm) [17]. The current study found no statistically significant differences between different methods of calliper placement when only measurements obtained in the same plane as the ultrasound probe are considered. The overall intra-observer reproducibility found in the current study are similar to those previously reported [16, 18].

The mean difference between AAA diameter measured by the ITI, LETLE and OTO methods were comparable to those previously reported and relate to vessel wall thickness [16, 17]. These differences highlight the importance of having clearly defined methods of calliper placement that are consistently used in both clinical practice and research. Multiple studies have looked at the influence of using alternative methods of calliper placement on the recruitment of patients into surveillance programmes. ITI measurements underestimate AAA size and lead to reduced sensitivity when used as a screening tool. A previous study analysed the influence of calliper placement on AAA prevalence in a cohort of 18,698 patients and found that it led to a significant difference in AAA diagnosis and subsequent recruitment into surveillance programmes (AAA prevalence ITI = 3.3%, LETLE = 4.0% and OTO = 5.9%) [16].

This study suggests that the ITI, OTO and LETE calliper placement methods can be equally well reproduced when placed in the same plane as the US probe, i.e. anterior–posterior. The measurement of transverse ITI or OTO diameter is not as reproducible. These findings suggest that measurement in the anterior–posterior plane should be used in clinical practice and clinical trials. Since the repeatability of measurements is much better within rather than between individuals it is also preferable for measurements to be performed by the same observer.

The current study used modern ultrasound technology and standardised methodology to directly compare the three leading methods of calliper placement. Of the 16 previous reproducibility studies identified seven [23, 27, 29–33] were published prior to 2000 and only three [16–18] reported the inter-observer reproducibility for all three methods of calliper placement and two [16, 17] the intra-observer reproducibility. This study examined the variation in measurements introduced by different methods of calliper placement on static AAA images obtained at a single time point. Measurement error introduced during the acquisition of scans was not assessed and therefore in clinical practice the reproducibility coefficients are likely larger.

## Conclusions

In conclusion, AAA diameter measurements obtained perpendicular to the orientation of the ultrasound probe (anterior–posterior) can be performed more reproducibly than those performed in the transverse plane. Measurements performed by the same observer also have better repeatability than those performed by different observers. The findings suggest that measurements of AAA size should be performed in the anterior–posterior plane and compared between different time periods by the same observer, particularly for situations such as clinical trials where high precision is required to sensitively detect changes in AAA diameter over time.

## Data Availability

All data generated or analysed during this study are included in this published article.

## Abbreviations

AAA: Abdominal aortic aneurysm
AP: Anterior–posterior anatomical plane of the body
TV: Transverse plane of the body
ITI: Inner-to-inner walls of the aorta
OTO: Outer-to-outer walls of the aorta
LETLE: Leading edge-to-leading edge within the walls of the aortas
LOA: Limits of agreement
